# A diverse mammal-dominated, footprint assemblage from wetland deposits in the Lower Cretaceous of Maryland

**DOI:** 10.1038/s41598-017-18619-w

**Published:** 2018-01-31

**Authors:** Ray Stanford, Martin G. Lockley, Compton Tucker, Stephen Godfrey, Sheila M. Stanford

**Affiliations:** 1 NASA/Goddard Space Flight Center, Greenbelt, Maryland 20771 USA; 2 Dinosaur Trackers Research Group, Campus Box 172, University of Colorado Denver, PO Box 173364, Denver Colorado, 80217-3364 USA; 3 Earth Science Division Code 610.9, NASA/Goddard Space Flight Center, Greenbelt, Maryland 20771 USA; 4 Calvert Marine Museum, 14200 Solomons Island Road, Solomons, Maryland 20688 USA, and National Museum of Natural History, Smithsonian Institution, Washington, DC 20560 USA

## Abstract

A newly discovered assemblage of predominantly small tracks from the Cretaceous Patuxent Formation at NASA’s Goddard Space Flight Center, Maryland, reveals one of the highest track densities and diversities ever reported (~70 tracks, representing at least eight morphotypes from an area of only ~2 m^2^). The assemblage is dominated by small mammal tracks including the new ichnotxon *Sederipes goddardensis*, indicating sitting postures. Small crow-sized theropod trackways, the first from this unit, indicate social trackmakers and suggest slow-paced foraging behavior. Tracks of pterosaurs, and other small vertebrates suggest activity on an organic-rich substrate. Large well-preserved sauropod and nodosaurs tracks indicate the presence of large dinosaurs. The Patuxent Formation together with the recently reported Angolan assemblage comprise the world’s two largest Mesozoic mammal footprint assemblages. The high density of footprint registration at the NASA site indicates special preservational and taphonomic conditions. These include early, penecontemporaneous deposition of siderite in organic rich, reducing wetland settings where even the flesh of body fossils can be mummified. Thus, the track-rich ironstone substrates of the Patuxent Formation, appear to preserve a unique vertebrate ichnofacies, with associated, exceptionally-preserved body fossil remains for which there are currently no other similar examples preserved in the fossil record.

## Introduction

Reports of true Mammalia tracks, from the Mesozoic, as distinct from tracks of presumed synapsids (therapsids) from early Mesozoic (mostly Triassic and Jurassic) dune facies, are rare, and mostly involve very small samples of isolated tracks. *Ameghichnus* isp. from the Jurassic of South America^1^ is the only convincing example of a pre-Cretaceous mammalian track^2–4^, later reported from isolated occurrences in North America^5^ and Europe^6^. The affinity of small mammaliform tracks^7,8^ from the Triassic-Jurassic transition in southern Africa is uncertain and compromised by problematic descriptions and access to original material. Indeed, the “lack of well-authenticated true mammal tracks from the Mesozoic is an impediment to interpretation of ichnofaunas”^9^.

The record of Cretaceous mammalian tracks is equally sparse, although slightly improved in recent years. *Koreasaltipes* isp., representing a small, mouse-sized Early Cretaceous hopping mammal, is the only example of an unambiguous trackway configuration^10^ which contrasts with *Schadipes* isp.^11^ the only other named ichnotaxon, preserved in an ambiguous trackway configuration. All other Cretaceous reports pertain to isolated tracks from the Aptian of Maryland^12^, isolated tracks from Tunisia^13^ and Angola^14–17^ and Colorado^18^. Of these only the Angolan tracks have been formally named as an ichnospecies within the ichnogenus *Catocapes*. An isolated specimen from Canada claimed as a syndactylous marsupial track^19^, was reinterpreted as an invertebrate trace^20^! Another purported mammal track from Canada^21^ was also dismissed as a misidentification^20^. Except for *Koreasaltipes* isp. and *Schadipes* isp., this sparse record does not allow confident discrimination between left and right tracks, between manus and pes, recognition of associated manus-pes sets, trackway segments, or confidence in formally naming tracks. Although body fossils of Cretaceous mammals (mostly teeth and jaws) outnumber tracks, with the exception of a few Chinese specimens, noted below, vanishingly few reveal foot skeletons.

Here we describe a remarkable, newly-discovered assemblage of Cretaceous tracks from the Patuxent Formation of Maryland discovered by the senior author. The assemblage from the Goddard Space Flight Center (GSFC-VP1) yields a diverse, high-density ichnofauna of ~70 dinosaur, pterosaur, mammal and indeterminate tracks: 26 attributable to mammals with diverse footprint morphologies, some in trackway configurations.

### Sedimentary geology of track-bearing units

**Figure 1:**
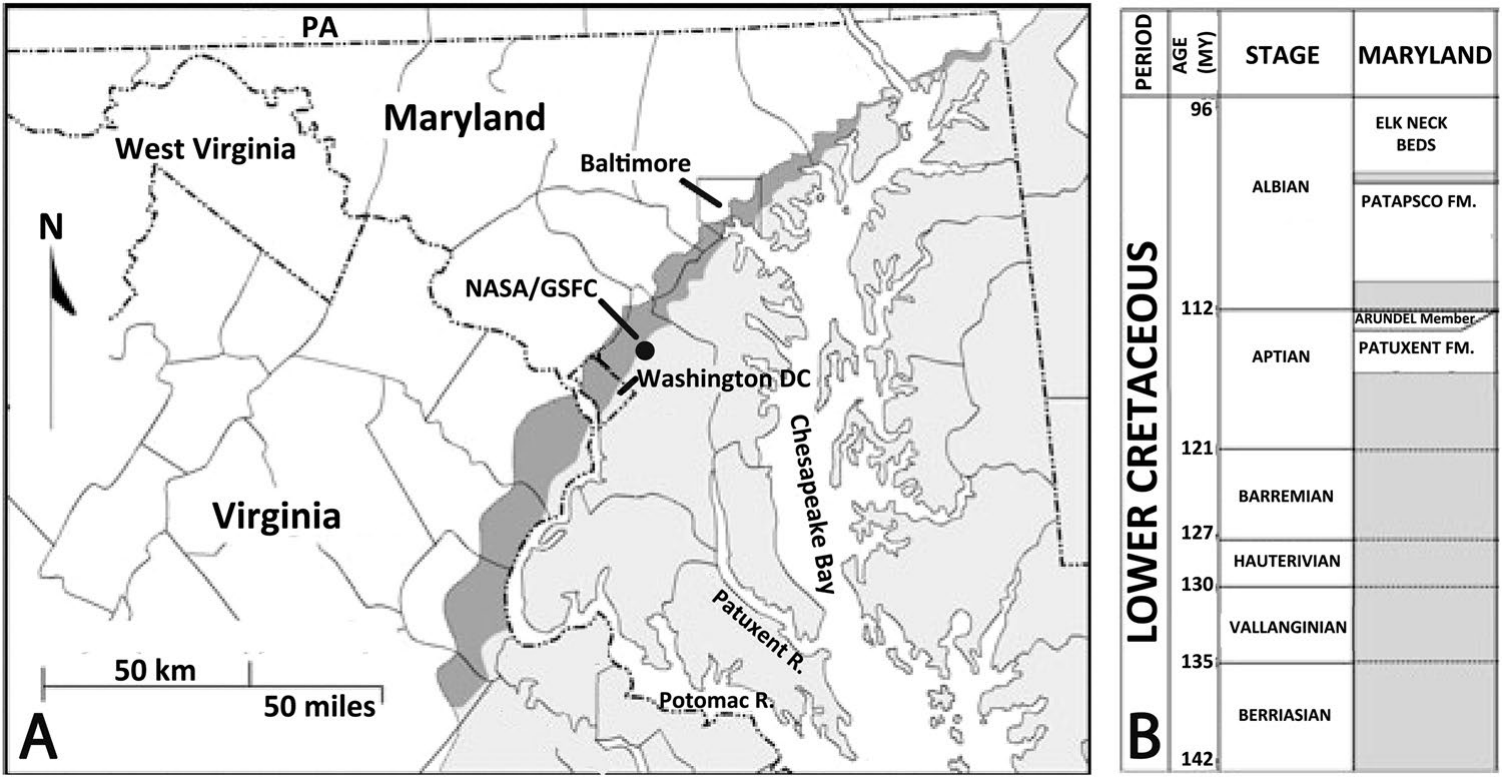
(**A**) Location of NASA Goddard Space Flight Center (GSFC) tracksite discovery in relation to outcrop of Patuxent Formation outcrop (dark gray) and younger overlying rocks (light gray). (**B**) Lower Cretaceous stratigraphy of study area. Modified after original maps compiled by authors and used by Stanford *et al*.^11,21^.

The ichnological potential of the Aptian age, Patuxent Formation, part of the Potomac Group, the oldest stratigraphic unit exposed in the Atlantic Coastal Plain region of Maryland and Virginia (Fig. 1) was first recognized in 2004^12^ with the discovery of a diverse assemblage of tetrapod tracks with unusual preservation^12,22^. The Patuxent Formation in Maryland, “is dominated by medium to coarse sands, sandstones, and pebble conglomerates … interbedded with large quantities of iron-cemented fine sandstones (formerly mined for iron), siltstones, and carbonaceous clays”^12^. Locally the large iron-cemented nodular concretions erode out as resistant slabs, to reveal tracks, as well as body fossil impressions.

The exquisitely-preserved impression of the anterior half of an articulated baby nodosaurian dinosaur (*Propanoplosaurus marylandicus*)^23^, with integument traces, discovered by the senior author, is evidence of exceptional preservation conditions. These helped preserve small tracks^12,22^ as well as body fossil impressions, including traces of integument, described as “desiccated dermal or fleshy elements … in fine grained siderite-cemented sandstone upon a thin (1–4 mm) layer of deep-red claystone” (Stanford *et al*., 2011, p. 917)^23^. In short, paleoenvironmental conditions preserved an articulated carcass or mummy with a full body length of about 30 cm.

Siderite-cemented surfaces are associated with hiatuses or “unconformities … commonly marked by ferruginous layers ranging from thin crusts to zones several inches in thickness … developed by weathering during the hiatus represented by the unconformity and as such penecontemporaneous in origin” (Glaser, 1969, p. 61)^24^. It is axiomatic that tracks are registered during hiatus phases in deposition. Thus, ironstone zones formed penecontemporaneously during such hiatuses helped create surfaces suitable for track registration. As noted in the study of *P*. *marylandicus* ^23^, “the precipitation of siderite requires a strongly reducing paleoenvironment” preserving abundant organic matter, and reducing conditions conducive to the flow of soluble iron in wetland environments^25^ and accounting for corpse mummification in bog or swamp settings^26^, as in the case of some material described here.

Previous studies^22^ indicate that the formation of ironstone zones facilitated the reworking of such resistant crusts or rinds into penecontemporaneous Lower Cretaceous clastic sediments that remained buried until exhumed by present day erosion^12,22^. The GSFC slab, however, is a large *in situ* deposit, not a reworked clast.

## The Patuxent trackways: description and context of the GSFC specimen

**Figure 2:**
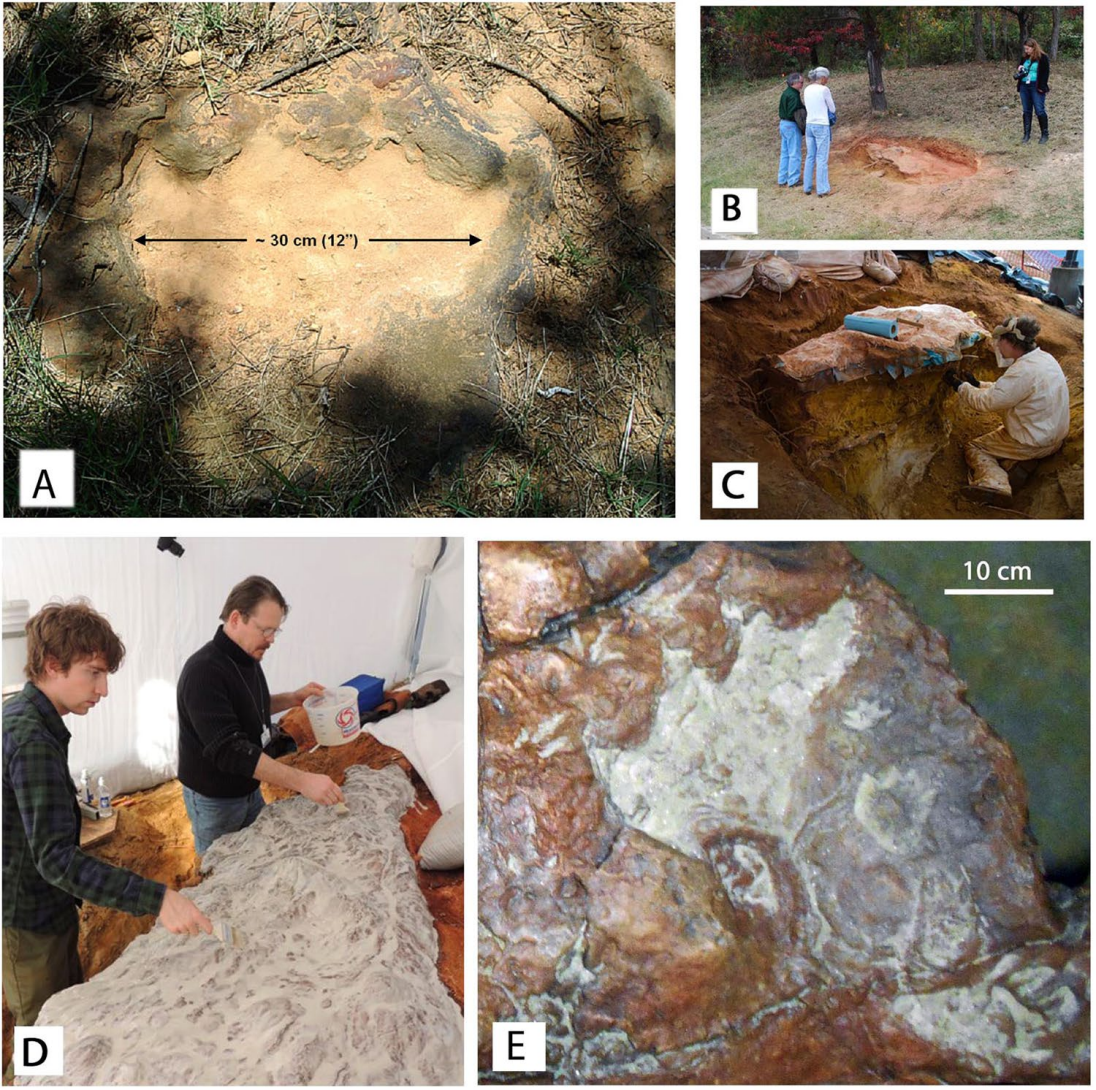
(**A**) “Discovery track” at time of discovery, (**B**) track-bearing slab *in situ* soon after discovery, (**C**) track bearing slab during excavation and jacketing. (**D**) The “discovery track” after replication, with small tracks around it. See Fig. 3 for orientation of the slab and Suppl info S1 for details of excavation. All photographs taken and compiled by the authors in Adobe Photoshop SC6.

**Figure 3:**
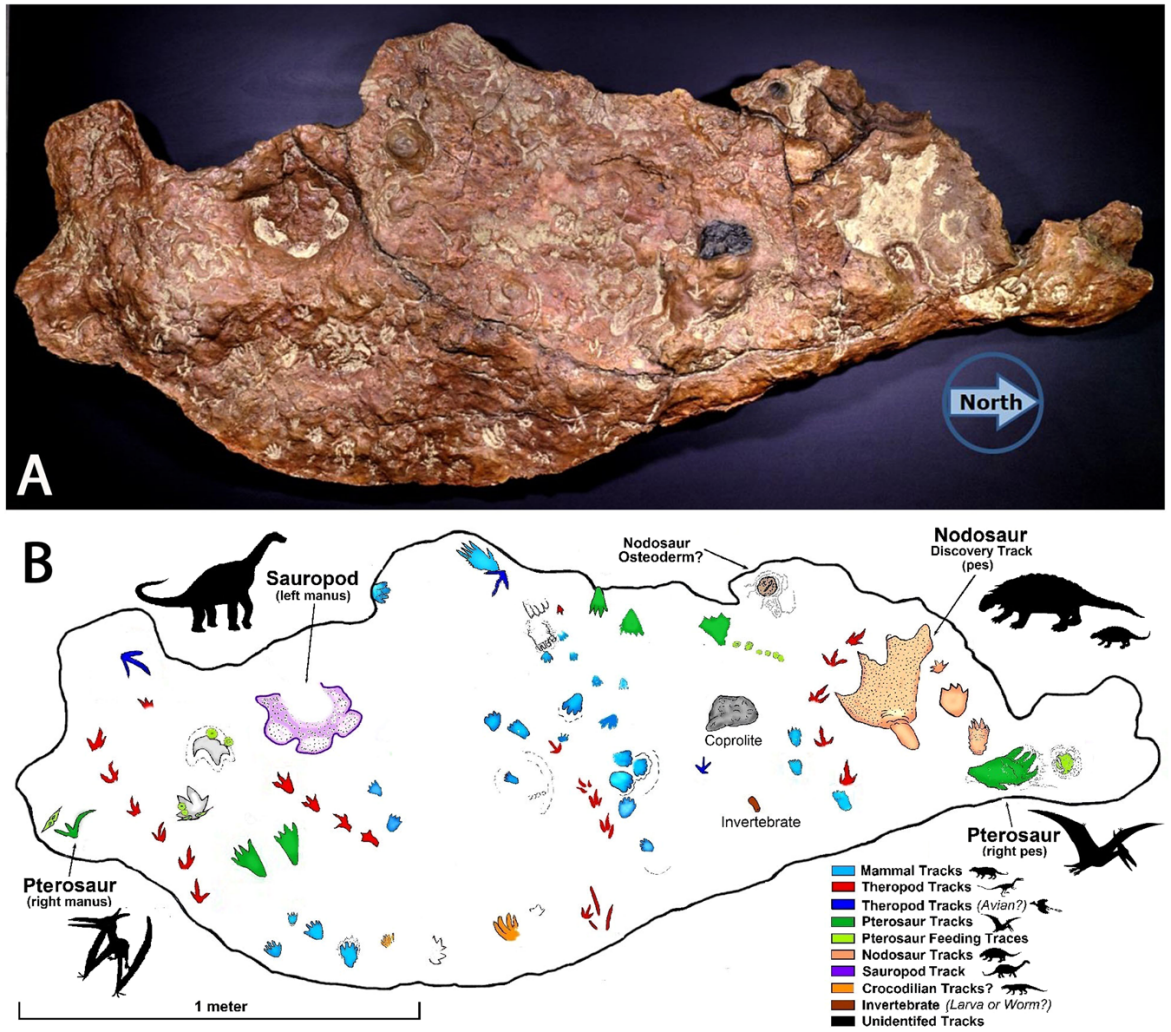
Photograph (**A**) and map (**B**) of replica GSFC-VP1 of whole track bearing surface. Note key to diversity of track morphotypes, and easily recognizable trackways of small theropods (in red), mammals (in blue) and pterosaurs (in green). The large discovery track, of inferred nodosaurian affinity, is situated beside small tracks also interpreted as nodosaurian. Track numbers corresponding to data are given in Supplementary Tables SI 1 and SI 2. See text for further details. All photographs and original map taken and compiled by the authors in Adobe Photoshop SC6.

We here describe the GSFC-VP1 specimen and relate the assemblage to previous track reports from the Patuxent Formation^12,22^. The track-bearing surface has very irregular topography (Figs 2–3). Large tracks up about 10 cm deep helped the senior author identify the “discovery track” in outcrop. By contrast 60–70 small tracks and other bioturbation features display relief of only a few millimeters. Conspicuous features of the surface include many wrinkles and tubercle- or bubble-like topographic features, which give the appearance of a solidified mud or gel. The excavation of the specimen described here as GSFC-VP1, is detailed in Supplementary Information SI 1.

While morphologically diagnostic ichnotaxa are attributed with varying degrees of confidence to trackmakers at higher, ordinal or familial levels, trackmakers are rarely inferred at the genus or species level. Thus tracks may be morphologically described, while their trackmaker attribution remains unknown or ambiguous. Understanding the GSFC-VP1 specimen benefits from previously-published, illustrated reports of isolated tracks from the Patuxent Formation^12,22^. Conversely however, the small size of previously described specimens, prevented study of continuous trackway segments or the association of different track types on large surfaces. The present study provides trackway information for several morphotypes that was previously unavailable.

93% of the previously identified Patuxent tracks^12^ had footprint lengths less than 18 cm and originated from isolated, reworked ironstone clasts. These were preserved as natural molds (concave epireliefs), casts (convex hyporeliefs), and sub-horizontal cross sections of abraded ironstone clasts: Stanford *et al*., (2007, Figs 13, 14 and 3A,B respectively)^12^. By contrast the GSFC VP1 specimen represents an *in situ* surface larger than any previously discovered. All tracks are natural impressions (concave epireliefs), and we recognize the first unambiguous Patuxent examples of continuous theropod trackway segments. Overall at least eight different track types are preserved representing dinosaurs, pterosaurs and mammals. These are described below using the taxonomic categories shown in Table 1.

**Table 1:** Ten general track morphotype categories represented in the Patuxent Formation based on the GSFC- VP1 specimen, and previous finds from the same formation.

Archosaurs	
Saurischian dinosaurs	
small-sized theropods	cf. *Grallator*
medium theropods	
sauropods	cf. *Brontopodus*
Ornithischian dinosaurs	
Ankylosaurid	cf. *Tetrapodosaurus*
Iguanodontid*	
Hysilophodontid*	*Hypsilophichnus* isp.
Pterosaurs	cf. *Pteraichnus*
*Mammalia*	
Morphotype A *Sederipes goddarensis*	new ichnospecies
Morphotype B	
Morphotype C	potential new ichnotaxon

Note that all categories are represented on GSFC-VP1 except medium theropods (*), iguanodontid (*) and *Hypsilophichnus* isp. (*), which were previously-named on the basis of material from other localities^12,22^.

### Small theropod tracks and trackways

**Figure 4:**
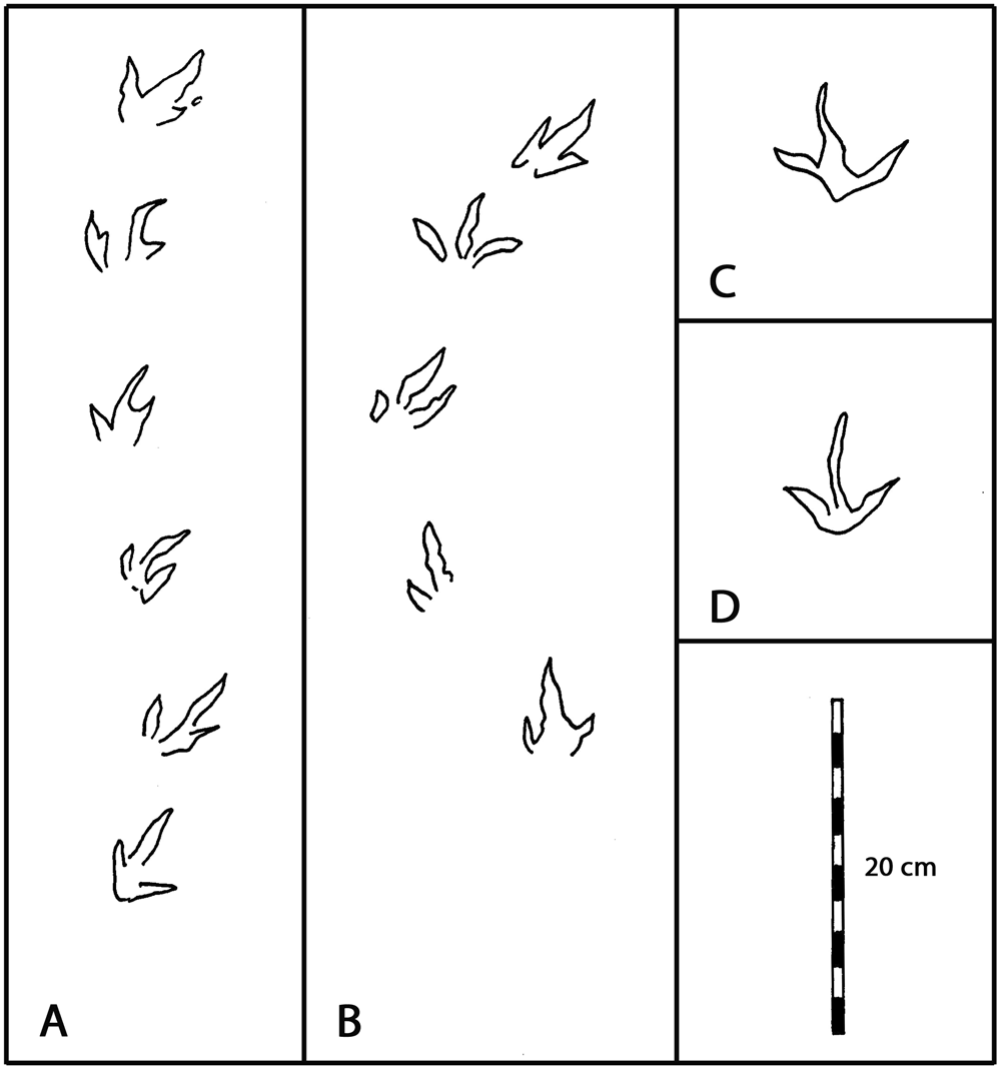
(**A**) Theropod trackway T1 with six footprints (T1.1-T1.6), (**B**) Theropod trackway T4 with five footprints (T4.1-T4.5). A and B both indicate very short steps. (**C**,**D**) Isolated tracks with wide digit divarication. Compare with Fig. 3B and Supplementary Fig. SI 1. Note that all tracks in T1 show rotation of middle digit (III) to right. Illustration compiled by the authors from original tracings and in Adobe Photoshop SC6.

Four small theropod trackway segments each reveal between four and six small, three-toed, moderately mesaxonic (tridactyl) tracks (Fig. 4). We provisionally label these cf. *Grallator* isp. Track size, shape and step (gait) are remarkably consistent: e.g., mean footprint length 4.64–5.67 cm, step 8.9–9.8 cm for four trackways (Table S1.1). Despite some curvature in trackway T1, T3 and T4 all are oriented more or less to the west with trackway T3 oriented to the southwest. Step measurements indicate very slow speed progression (0.75–0.80 km/hour: Table S1.1). The similarity in size, shape, step, stride and quality of preservation indicates the passage of similar-sized animals at the same time. The unusual right-side rotation of tracks in trackway T1 suggests an atypical, ‘sidling’ gait, perhaps caused by the animal looking to the right as it walked (Fig. 4A).

### Sauropod tracks

One large sauropod track (Fig. 3) with five distinctive digit traces is interpreted as a left front (i.e., manus) footprint. The overall sub circular shape is generally diagnostic for sauropods as is the closely bundled, equidimensional five toed (pentadactyl) morphology, with digit I having registered a sharper claw-like trace which contrasts with the blunt traces of digits II-IV. The track length (L) is 16.3 cm and the width (W) is ~25.7 cm. The traces of individual digits are unusually well preserved and indicate a columnar, digitigrade manus with blunt unguals. Such well-defined manus digit traces are rarely preserved, but have been recorded in the case of Lower Cretaceous *Brontopodus pentadactylus* from Korea^27^.

### Ornithischian track

The “discovery track” (Figs 2–3) is triangular in shape with four short but relatively pointed triangular digits. However, the posterior (heel) region is obscured by a smaller track of uncertain affinity. We infer the discovery track (n1) represents a nodosaurian, with the shorter digit trace (left side in Fig. 3) representing digit I of the right pes. The track is wider (FW ~29.0 cm) than long (FL ~22.0 cm). Nodosaurs have a pentadactyl manus, with the traces of digit I most prominent, and those of digit V least prominent^12^ whereas the pes is typically tetradactyl, and usually longer than wide: FL > FW, often with longer, more clearly-defined digit traces. Thus, we interpret the discovery track as a distorted hind footprint (pes). Tracks here labelled n2 and n3 respectively consist of a larger elongate 4-toed pes with a much smaller, anteriorly situated, transverse 5-toed manus. The manus-pes size difference indicates a high heteropody index. These likely also represent a nodosaurian or other ornithischian. If this interpretation is accepted, this inferred manus-pes set is the smallest yet attributed to an ankylosaurian, with a pes length and width of only 8.1 and 7.8 cm respectively (L/W = 1.04) and manus length and width of 2.9 and 4.9 cm respectively (L/W 0.59). Consistent with this interpretation several “ankylosaurian” manus tracks only 3.0–4.0 cm long were previously reported from the Patuxent Formation^12^. The shortest pes digit (right side in Fig. 3B) suggests a left manus pes set. These dimensions are of the same order of magnitude as the pes and manus remains of *Propanplosaurus* sp.^21^ (manus width ~3.0 cm) and the sharp distal terminations of the pes digit traces seem to mirror the form of the ungual sheath inferred for this taxon.

### Pterosaur Tracks

**Figure 5:**
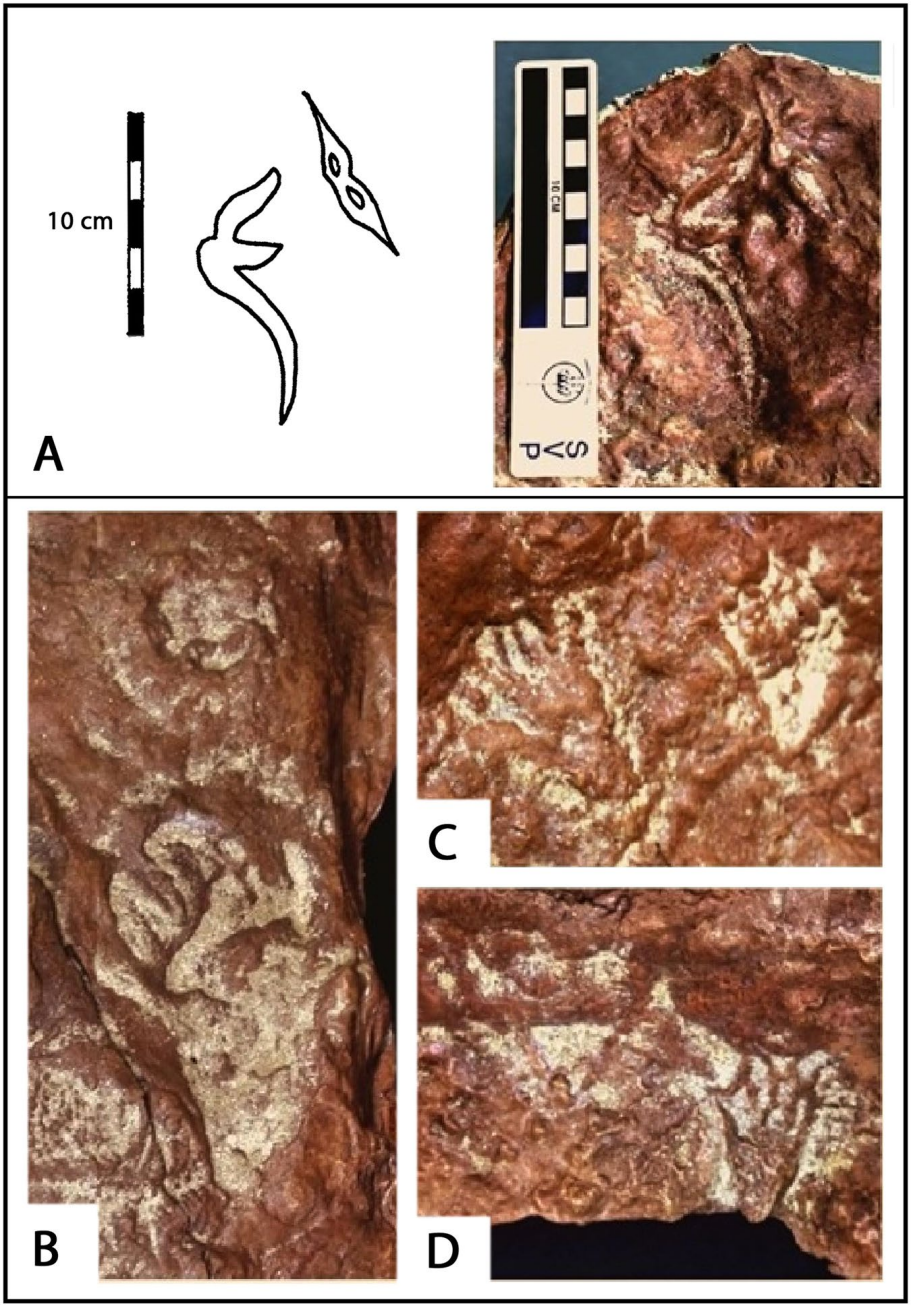
Pterosaur tracks. (**A**) Line drawing (right) and photo (left) of manus p1 with adjacent probable beak trace. (**B**) Large pes track p2 with probable beak trace, (**C**) two pes tracks p3 and p4. (**D**) Small pes track p5. All photographs and original tracing taken and compiled by the authors in Adobe Photoshop SC6.

At least one pterosaur manus track (p1, Fig. SI2.1) and four probable pes tracks (p2–p5, Fig. SI2.1) have been identified (Fig. 5). The manus track is ~12.0 cm long and 4 cm wide, and appears to be associated with a paired trace resembling a beak probe mark (Fig. 5A). The inferred pes tracks vary in length from about 7.0- 17.0 cm, with corresponding widths of ~4.0 and 9.0 cm, and the larger track may also be associated with beak probe marks (Fig. 5B). The size range is consistent with that of the sample of isolated pterosaur track reported previously^12^.

### Mammal Tracks

**Figure 6:**
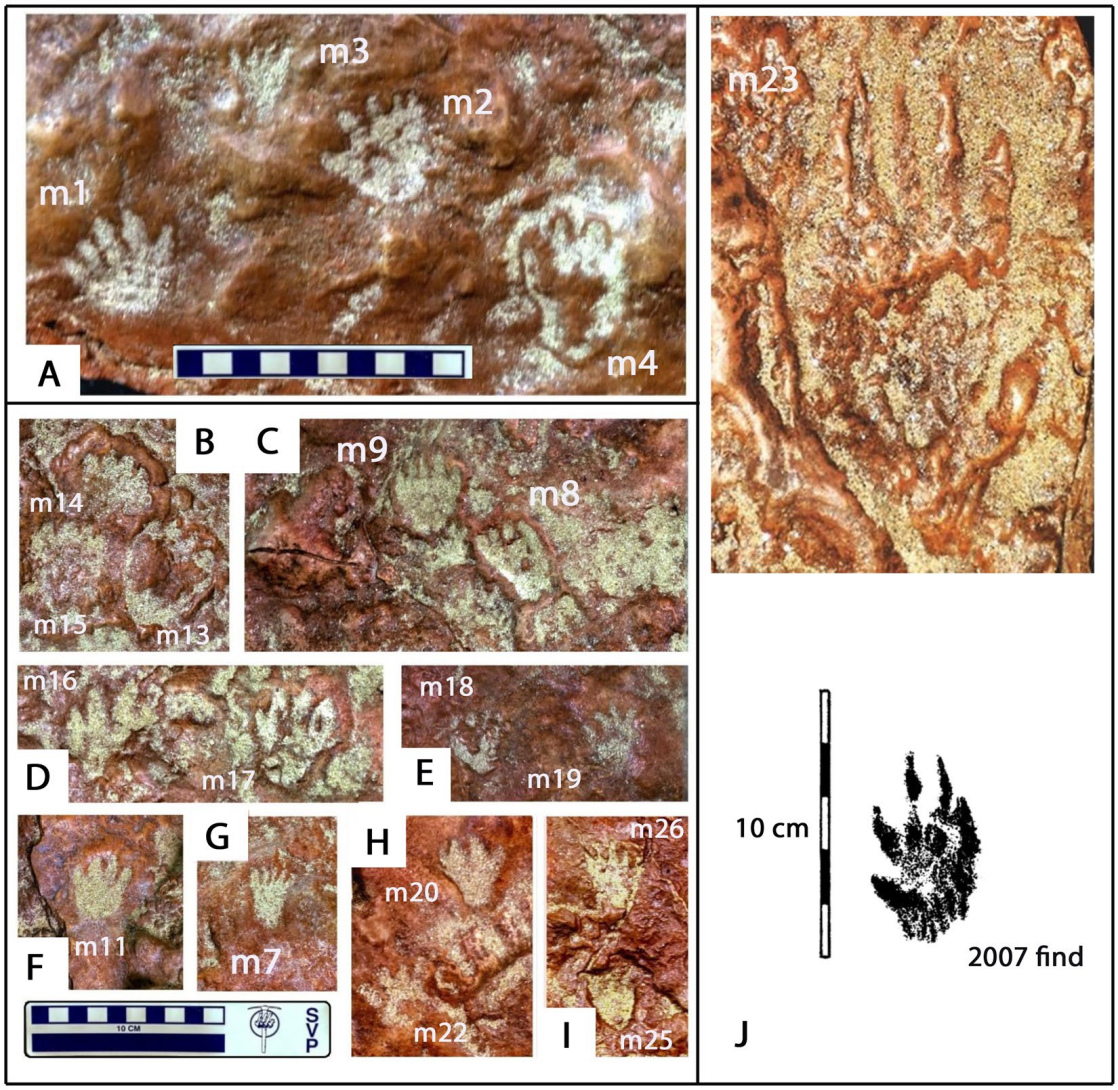
Mammal Tracks registered on GSFC specimen GSFC-VP1. (**A**) Tracks m1-m4 include m1-m2 holotype of *Sederpes goddardensis* a pentadactyl left –right pair (m1 and m2). (**B**) Tracks m13-m15 include m14 with pronounced anterior mud rim. (**C**) m8 and m9, (**D**) m16 and m17 probably represent a left right pair, (**E**) m18 and m19 represent a possible pair, (**F**) m11, (**G**) m7, an elongate pentadactyl track, (**H**) m20 and m22, (**I**) m25 and m26. (**J**) m23 large pentadactyl track (photo above) with image of similar track described in 2007^12^. Compare with Fig. 3 and SI Fig. 1. All photographs taken and compiled by the authors in Adobe Photoshop SC6.

**Figure 7:**
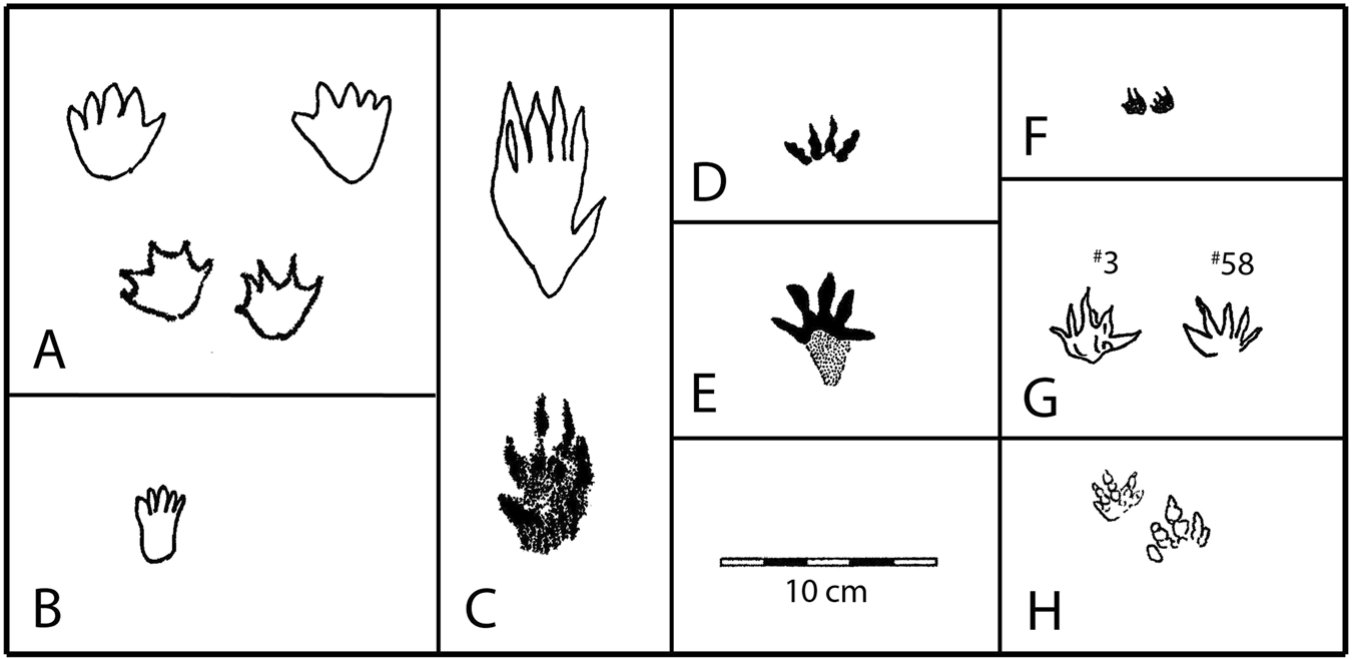
Maryland mammal tracks provisionally assigned to morphotypes (**A**–**C**). (**A**) *Sederipes goddardensis* holotype (top) preserved as pes pair with similar, smaller pes pair^12^, (**B**) Moprhotype B, with elongate heel trace, (**C**) Morphotype C, pentadactyl track with a large divergent posterior digit (top) and similar smaller track^12^, (**D**) *Schadipes crypticus* ^11^, and (**E**) unnamed morphotype from *Schadipes* type locality^38^, (**F**) *Koreasaltipes jinjuensis* holotype, (**G**) *Catocapes angolanus* holotype (#3) and paratype (#58)^17^, (**H**) unnamed mammal tracks from Tunisia^13^. Note all tracks are Cretaceous in age and drawn to same scale. Compare A–D with Fig. 6. All track outlines taken from original tracings and compiled by the authors in Adobe Photoshop SC6.

True mammalian tracks are rare in the Mesozoic, with only one named ichnotaxon (*Ameginichnus*) named from the Jurassic and three (*Schadipes* isp., *Koreasaltipes* isp. and *Catocapes* isp.) named from the Cretaceous: see discussion. A variety of mammal or mammaliform tracks were registered on the GSFC- VP1 specimen surface. We recognize 26 tracks (Figs 3, 6 and Supplementary Fig. SI 1 and Table SI 2) representing at least three morphotypes (Fig. 7), distinguished on the basis of size and morphology. As mammal tracks, representing eutherians and/or metatherians, are rare in the Mesozoic, there is little precedent for identifying them or assigning taxonomic labels. Mesozoic mammal footprints are rarely preserved in trackways and those that are (*Ameginichnus* isp., *Schadipes* isp. and *Koreasaltipes* isp.) indicate hopping gaits. It is impossible to speculate on the gaits of the trackmakers of isolated tracks. However, the Patuxent sample contains a few examples of paired tracks (here named *Sederipes goddardensis*) which indicate the right and left hind feet in a sitting position. In addition to a pair of pentadactyl tracks illustrated by Stanford *et al*. 2007 (Fig. 17A)^12^, but not discussed in detail, we identify another pair (m1 and m2: Fig. 6A) which indicate this behavioral posture, which is also inferred for the pairs m13 and m14 (Fig. 6B), m16 and m17 (Fig. 6D) and possibly for m18 and m19 (Fig. 6E).

### Morphotype A

At least three pairs of tracks assigned to Morphotype A have been found in symmetrical left and right configurations (m1 and m2, Fig. 6A; m13 and m14, Fig. 6B). Likewise the pair illustrated by Stanford *et al*. 2007 Fig. (17A)^12^ (Fig. 7A) resembles the well preserved pair (track m1 and track m2) which show an almost perfect mirror image morphology with the inner digit (I) shorter than the others (II-V), a diagnostic crown mammalian pattern^28^. A pronounced sediment rim around track m14 highlights a similar pentadactyl morphology. Based on the pair m1 and m2 and the aforementioned pair^12^ (Fig. 7A) the tracks are about as wide as long (L/W ratio ~1.0). Track lengths may be exaggerated by forward motion, but in all cases track width is between ~4.0 and ~5.0 cm. The sitting-on-haunches posture appears to be typical of the Patuxent sample, and justifies the naming of a new ichnotaxon (*Sederipes goddardensis*) based on a previously unreported ichnological register of a posture (behavior) diagnostic of small mammals: see systematic section and Supplementary Information.

### Morphotype B

Morphotype B as represented by track m7 (Figs 6F and 7B) is an isolated, elongate, pentadactyl track 3.6 cm long and 2.6 cm wide (L/W ratio 1.38). A number of other tracks (e.g., m3 and m26) appear similar. In such small tracks it may be difficult to discern the shorter digit (I) which may make the track appear tetradctyl (e.g. m11, m19).

### Morphotype C

A single, large, five-toed (pentadactyl) track (m23 of Supplementary Fig. SI 1) has a distinctive morphology with a short posterolateral “digit” (digit I), and four equidimensional digits (II-V). It is ~11.4 cm long and ~5.9 cm wide. The morphology of m23 bears a striking resemblance to the smaller (FL ~7.0 cm) isolated track described and illustrated by Stanford *et al*. (Fig. 16, and Figs 6I and 7C herein)^12^, except the relative lengths of digits II-V are somewhat different. The smaller Morphotype C track has distinct pad impressions resembling those of the extant musk rat and other modern rodents (SI3, Fig. 3.1A,B). As this morphotype has not previously been reported from the Mesozoic, it would warrant description as a new ichnospecies, if a trackway configuration were found. At first sight, this elongate, narrow heeled tetradactyl track resembles a pterosaur pes. But to date all known pterosaurian pes tracks are tetradactyl, not pentadactyl, which is the typical condition in Cretaceous pterodactyloids.

## Systematic ichnology

Three of the four previously named Mesozoic mammal ichnotaxa have been based on trackways. *Ameghinichnus* isp. from the Jurassic of Argentina^4^, *Koreasaltipes* isp. from the Early Cretaceous of Korea^9^ and *Schadipes* isp. from the Late Cretaceous of Colorado^11^ all indicate hopping gaits. Isolated tracks from the Cretaceous of Tunisia^13^ and Angola^14–17^, as well as some from Colorado^18^ and Maryland represent unknown locomotor gaits, and are not appropriate as the basis of new ichnotaxa^29^, unless isolated footprint morphologies are highly distinctive and unique, as is potentially the case with Morphotype C (described above), and as argued below for Morphotype A. In this regard, pentadactyl tracks from the Cretaceous of Angola were named as *Catocapes angolanus* on the basis of an isolated holotype and paratype^17^: see Fig. 7G. The utility of this ichnotaxon is debatable, especially as it is based on type material that cannot be identified as pes or manus! Due to equal uncertainty about manus-pes differentiation pertaining to the recently described track from Colorado^18^, it was not named. This dilemma generally pertains to all isolated tracks.

The pairs of tracks described here as *Sederipes goddardensis*, occur in symmetrical pairs, but not long trackway sequences. They suggest a temporarily “sedentary” sitting posture, with hind feet situated as mirror images on either side of the animal’s parasagittal plane. Such sitting postures are characteristic of extant small mammals such as mice and squirrels, as well as some small insectivores and carnivores which almost invariably have front limbs and hands which are much smaller than hind limbs and feet, often held off the ground. (Suppl. Info). From an ichnological and behavioral viewpoint a bipedal posture including sitting phases represents a ‘complete’ ichnological expression of trackmaker posture conforming to the guidelines for naming new ichnotaxa^29^. To date, traces indicating a sitting, or sitting-on-haunches posture, have not previously been reported for class Mammalia, although they are known for squatting or crouching dinosaurs (Suppl. Info).

### Class Mammalia

*Sederipes* ichnogenus nov.

#### Diagnosis

Small pentadactyl pes tracks with digit I trace shorter than equidimensional traces digits II-V arranged parasagittally, in symmetrical pairs indicative of sitting posture.

**Holotype**: pair of tracks designated as m1 and m2 on specimen GSFC-VP1.

**Paratypes**: pairs of tracks designated as m13 and m14 on specimen. Pair of tracks illustrated by Stanford *et al*., (2007, Fig. 17A)^12^.

**Derivation of ichnogenus name**: From Latin *sedere* “to be in a sitting position” and pes meaning foot.

**Type horizon and locality**: NASA/Goddard Space Flight Center, Maryland, USA.

*Sederipes goddarensis* ichnospecies nov.

**Diagnosis**, as for ichnogenus.

**Description**: Small pentadactyl pes tracks, about as wide as long, with digit I shorter, about half length of longer equidimensional traces of digits II-V. Digit I separated from digit II by wider hypex than hypices between digits II-III, III-IV and IV-V. Digits II-IV about half length of track, with posterior half of track an undifferentiated pad trace with broad, sub-circular, posteriorly-convex margin to heel. Tracks arranged in symmetrical pairs indicative of sitting posture.

**Holotypes, paratypes and type horizon and locality**: as for ichnogenus.

**Derivation of ichnospecies name**: *goddardensis* from Goddard Space Flight Center.

## Body Fossils

**Figure 8:**
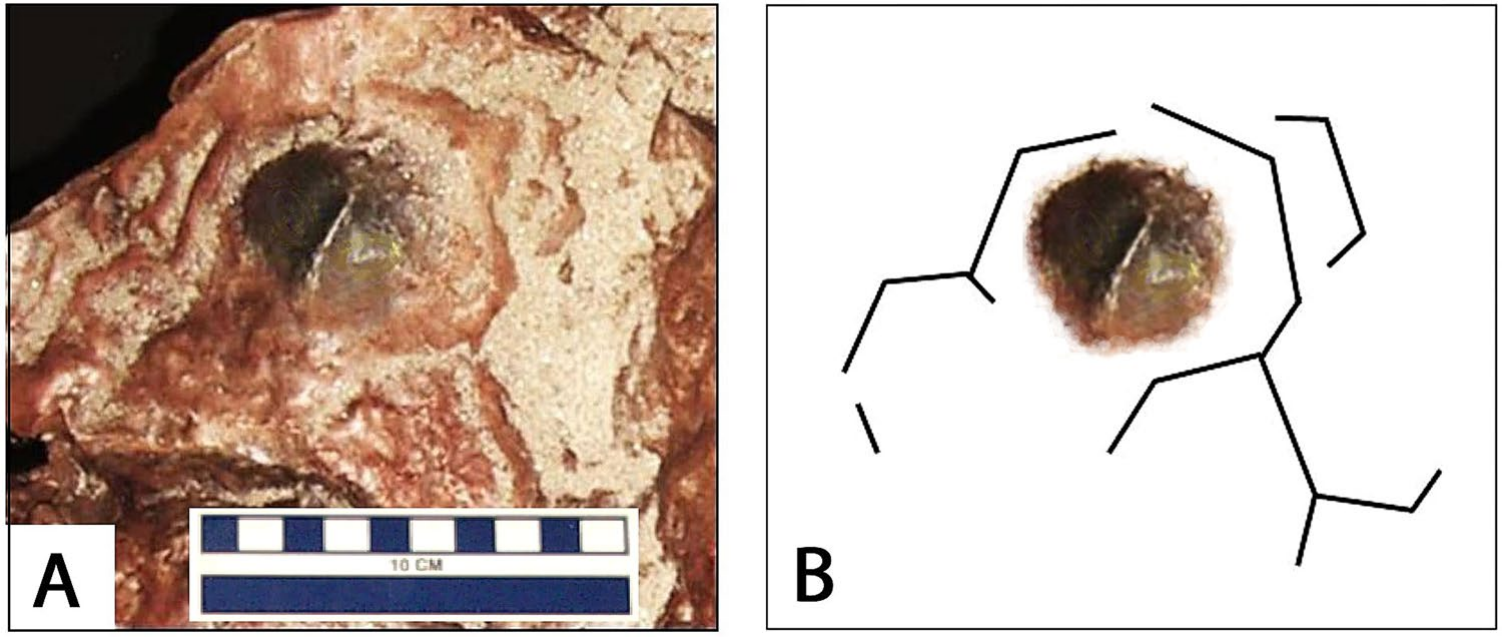
(**A**) Photograph of nodosaur scute and associated polygonal pattern of surrounding integument, (**B**) simplified outline of polygonal pattern. All photographs taken and compiled by the authors in Adobe Photoshop SC6.

One of two dark gray to black features on the GSFC-VP1 specimen surface (Fig. 3A) is interpreted as a nodosaurian osteoderm (Fig. 8). The specimen, ~5.0 cm in diameter with a pronounced central ridge, is morphologically similar to the isolated or articulated bony scutes found in association with nodosaurian skeletal remains. The scute is surrounded by what appear to be the margins of polygonal areas that ornament the integument. The scute is centered on one of the more complete polygons.

The occurrence of body fossil remains, attributable to a nodosaurian is entirely consistent with the discovery of the Patuxent baby nodosaur body fossil^23^. Prior to this study^12^ skin impressions with a smaller polygonal pattern (~1.0- 1.5 cm diameter) had been reported but not attributed to any particular dinosaurian group. They resemble sauropod skin traces.

## Discussion and Synthesis

Ichnofaunas allow raw counts of number of tracks, trackways and diversity of track morphotypes, thus, serving as proxy vertebrate diversity estimates. Lack of trackways^12,22^ forces us to estimate diversity on the basis of raw track counts (Table 2). We conservatively estimate 10 Patuxent track morphotypes (Table 2) of which at least eight occur on the GSFC-VP1 slab. These are theropods (2 morphotypes), sauropods, iguanodntids, hypsilophodontids, ankylosaurids, pterosaurs (2 morphotypes) and? three mammalian morphotypes. These eight GSFC morphotypes, representing an estimated 8/10^ths^ of the ichnofaunal diversity, occur on a surface of only about 2 m^2^, thus making this slab highly representative of the entire vertebrate ichnofauna. Therefore, the GSFC slab is the Patuxent Formation’s ichnological window (?Rosetta Stone) into Lower Cretaceous paleoecology of the region. The count of ~67 tracks represents one of the highest track densities known from the Mesozoic, and is also one of the highest diversities (Table 2). The recent report^17^ of an Angolan assemblage of ~70 small tracks (numbers 1–70) on a “chaotically trampled” surface, indicates a similar density. This study’s sketch map showed 42 of these numbered tracks (35 mammaliamorph and 7 crocodylomorph) in an area of about 1.0 × 1.6 m (1.60 m^2^), with an additional crocodylomorph trackway making the tracked area ~1.80 m^2^. 45 of the 70 Angolan tracks were interpreted as mammaliamorph, and four including the holotype were described as “the best preserved isolated tracks”^17^. However, no mammaliamorph trackway configurations were described from the Angolan sample, and manus and pes tracks were not distinguished, even in the case of the holotype (no. 3: Fig. 7G herein). This assemblage therefore is similar in size and track density to the GSFC assemblage, which was registered on an area of ~1.90 m^2^. Assuming the GSFC theropod trackways and several mammal track pairs or clusters (Figs 3 and 6) represent single individuals, we estimate the minimum number of trackmakers represented by the ~67 footprints as about 40 individuals representing about eight distinct taxa, registered about 67 identifiable footprints in an area of about 1.90 m^2^.

**Table 2:** Synthesis of trackway data from the present and previous studies^12,22^ of the Pautuxent ichnofauna, based on number of tracks and track morphotypes.

Track type all studies	No. tracks this study	Estimated no. trackways this study	% tracks GSFC specimen	% based on 100 tracks identified by Stanford *et al*.^12^	Total of tracks/% from combined sample present study & Stanford *et al*.^12^
small theropods	23	7	34	18	41 = 24.5%
medium theropods				14	14 = 8.4%
sauropods	1	1	1.5	10	11 = 6.6%
iguanodontid				10	10 = 6.0%
hypsilophodontid				9	9 = 5.3%
ankylosaurid	4	1	7.5	19	15 = 11.4%
pterosaur	5	4	7	15	20 = 12.0%
3 mammal morphs	26	20	38.9	10	36 = 21.5%
others	8	?7			8 = 4.8%
**Totals**	**67**	**40**		**100 tracks = 100%**	**167 = 100%**

The Angolan and Maryland track evidence suggest that small mammal tracks often occur in high density assemblages in small areas where trackmakers were locally active on substrates suitable for small track registration. Such high densities are also seen in one of the Colorado samples^11^ where at least 30 indeterminate very small (length < 2.0 cm) mammal tracks occur on a surface of no more than 0.15 m^2^. Tracks of other small or large vertebrates may also occur in such areas.

A previous Patuxent track morphotype census was based on 100 small, identifiable, but isolated specimens, (Table 2) mostly revealing only single tracks, and none with more than two track types^12^. The diversity estimate of about 14 trackmaker types was slightly higher than the estimate derived from the present study of the GSFC-VP1 slab due to the identification of medium sized theropods, two ornithopod morphotypes and possibly two pterosaur track morphotypes.

The census database based on previously-collected Patuxent samples^12^ of transported “float” material can be compared with GSFC VP1sample representing a single *in situ* assemblage. The latter data reveals a rather higher proportion of mammal (~39%) and small theropod tracks (34%). Nevertheless, although exact stratigraphic correlations between the assemblages are not known, the ironstone lithofacies are very similar and both assemblages are diverse and dominated by small tracks. Thus, the single *in situ* GSFC sample is almost as representative of the ichnofauna as the small samples. This, justifies combining both data sets for an overall census (N = 167) and estimating that tracks of small theropods (24.5%) and mammal (21.5%) make up almost half the entire ichnofauna (Table 2).

Repeat associations of particular ichnotaxa in given facies help define vertebrate ichnofacies and vertebrate ichnocoenoses^30,31^. The repeat association of Patuxent tracks in a distinctive ironstone ichnofacies suggests conditions favorable to the preservation of diverse assemblages of small tracks. Such occurrences mitigate widespread biases against the preservation of small tracks^32,33^.

Given that terrestrial vertebrate body fossils are rare in the Patuxent Formation (the occurrence of *Proplanoposaurus* notwithstanding)^23^, the ichnofauna assumes added importance in characterizing the paleoecology. The mammal tracks are particularly important, because so few are known from the global track record. Although mammal body fossils are moderately well known in North America, and elsewhere they are mostly represented by teeth and jaws, not foot skeletons. Fully articulated Cretaceous mammals are best known from the Yixian Formation in China^34–36^ and incomplete foot skeletal remains have also been found in Mongolia^37–39^. Thus, the potential to match Cretaceous footprints and foot skeletons is limited by small samples in both categories.

The entire record of Cretaceous mammal tracks is sparse, consisting, in order of discovery, of small samples from Colorado^11^, Maryland^12^, Tunisia^13^, Angola^14–17^ and Korea^10^ and again from Colorado^18^. Most of the tracks including formally named *Schadipes* isp. from Colorado and *Koreasaltipes* isp. are smaller than morphotypes A and C described here. The preliminary Angolan report^16^ described mammaliamorph, “functionally pentadactyl” tracks with “divergent central digits (II-IV) … and more divergent lateral digits (I and V) “ with the average length of 2.7 cm and width of 3.2 cm suggesting an animal “as big as a modern raccoon” and “comparable in size to *Repenomanus*” (Marzola, 2014a, p. 181)^14,15^. This interpretation was repeated in a more detailed study^17^ confirming the track size as averaging 2.7 cm long and 3.2 cm wide and claiming these as “the largest mammaliamorph tracks known from the Early Cretaceous unmatched in size in the skeletal fossil record”^17^. These assertions require re-evaluation and comment.

*Catocapes* isp. is indeed larger than *Koreasaltipes* isp. and about equal in size to unnamed tracks from the Cenomanian of Tunisia, technically Late Cretaceous in age^13^. *Catocapes* is also somewhat larger than Late Cretaceous (Campanian-Maastrichtian) *Schadipes* isp. from Colorado^10^, but it is smaller than another track from the *Schadipes* isp. locality^18^. Given that Patuxent Morphotype C is large (track lengths ~6–11 cm) and included a previously described specimen^12^, *Catocapes* is clearly not the largest Early Cretaceous mammalian track morphotype presently known. Morphotypes A (*Sederipes* isp.) and B also have track lengths and widths in the 4–6 cm range, and so are as large or larger than *Catocapes* isp., and Morphotype C is 2–3 times larger than any of the putative mammalian tracks reported from the Lower Cretaceous. If, as claimed^17^, there are no potential Early Cretaceous trackmakers capable of registering tracks 3.2 cm long, many of the Patuxent mammal track morphotypes represent animals much larger than any known from skeletal remains. Thus, the Patuxent track record, is even more suggestive of trackmakers much larger than *Repenomanus* sp., than are the comparatively small Angolan tracks.

The GSFC-VP1 assemblage, the first large *in situ* Patuxent ichnofauna allows us to make a proxy trackmaker census at a single site, and single instant in geological time. The census data confirms that previously accumulated from many small isolated samples. Thus, GSFC-VP 1 is a “key” window into the Early Cretaceous Patuxent paleoecology. The main features of this ichnofauna include an abundance of small mammals tracks representing at least three morphotypes, including the first repeat assemblages of paired pes tracks (*Sederipes goddardensis*) representing mammals in sitting postures. The sample also reveals the first reported, continuous multiple, subparallel trackways of small ‘crow-sized’ theropods, ostensibly engaged in slow speed movement, perhaps foraging as a “social” group on undulating, wetland terrain frequented by mammals, pterosaurs and bioturbating invertebrates. Integument remains, the sedimentological evidence and the high density of tracks, suggest the substrates were organic rich foraging grounds, for a diverse fauna.

The Patuxent ichnofauna represents a high diversity ironstone-wetland ichnocoenosis, or simply an “ironstone ichnocoenosis.” This ichnocoeosis does not obviously fit in the 5-fold Archetypal Tetrapod Ichnofacies scheme proposed by some authors^30^ where five ichnofacies are associated with: 1) eolian, 2) tidal flat-alluvial plain, 3) lacustrine margin, 4) the shallow lacustrine and 5) coastal plain paleoenvironments and characteristic tetrapod traces. The Patuxent “ironstone ichnocoenosis” might loosely be subsumed in the coastal plain ichnofacies which is purported to comprise a majority of large, terrestrial, quadrupedal herbivore tracks and few (>10%) terrestrial carnivore tracks^30^. The “ironstone ichnocoenosis” fits this description only in the most general way, if the small tracks of theropods (carnivores) and mammals (probably omnivores) are overlooked. This tells us small tracks are rarely registered in many coastal plain ichnofacies. Likewise, while Colorado’s Laramie Formation coastal plain deposits, rich in organic remains, may represent the large-herbivore-dominated, coastal plain ichnofacies, the small theropod and mammal tracks^11,18^ are reminiscent of the Patuxent ichnofaunas. In short, small tracks may help better reassess ichnofacies characteristics.

Definition of vertebrate (tetrapod) ichnofacies are complex (Suppl. Info^40–50^). It is nevertheless uncontroversial to note that small tracks are often underrepresented^32,33^, due to suboptimal preservation. Thus, bias towards preservation and recognition of large tracks affects ichnofacies definitions. Colorado’s Laramie Formation indicates “intermediate” substrate and taphonomic conditions where some small tracks are preserved. By contrast, the Patuxent ichnocoenosis represents near-optimal conditions for the preservation of small tracks. It is “a window” on the coastal plain paleoecology of the Maryland region, more nearly representing an optimally preserved ichnofacies fauna, and also far more informative than the impoverished body fossil record.

### Data availability statement

All data compiled in this study is available in the submitted manuscript and supplementary information and from the authors.

## Electronic supplementary material

Supplementary Information

## References

[1] Casamiquela, R. M. *Estudios Icnologicos*. Colegio Industrial Pio IX 229, Buenos Aires, 26 p. (1964).

[2] Leonardi, G. Annotated Atlas of South American Tetrapod Footprints. Ministerio de Minas e Energia, Republica Federativa do Brasil. 247 (1994).

[3] Rainforth, E. C., & Lockley, M. G. Tracks of diminutive dinosaurs and hopping mammals from the Jurassic of North and South America in *Continental Jurassic Symposium Volum*e(ed. Morales, M.) 265–269, Museum of Northern Arizona (1996).

[4] de Valais, S. Ichnotaxonomic revision of *Ameghinichnus*, a mammalian ichnogenus from the Middle Jurassic La Matilde Formation, *2203Zootaxa*, Santa Cruz province, Argentina 1–21 (2009).

[5] Szajna, M. J. & Silvestri, S. M. New occurrences of ichnogenus *Brachychirotherium*: implications for Triassic-Jurassic mass extinction events, in Morales, M. (ed). The Continental Jurassic. Bulletin of the Museum of Northern Arizona **60**, 275–283 (1996).

[6] Gierlinski GD, Niedzwiedski G, Pienkowski G (2004). Tetrapod trackl assemblages in the Hettanghian of Slotykow, Poland. Ichnos.

[7] Ellenberger, P. Contribution a la classification des pistes de vertebres du Trias; les types du Stormberg d’Afrique du Sud, 2e partie. *Palaeovertebrata Memoire extraordinaire*, Montpellier 170 (1974).

[8] Ellenberger P (1975). L’explosion demographique des petities quadrupedes a l’alure de mammiferes dans le stormberg Superieur (Trias) d’Afrique du sud apercu sur leur origine au Permien (France et Karoo). Int. Col. Nat. Center Sci. Res. Bull..

[9] Lockley MG, Lucas SG, Gaston R, Hunt AP (2004). Ichnofaunas from the Triassic-Jurassic boundary sequences of the Gateway area, Western Colorado: Implications for faunal composition and correlations with other areas. Ichnos.

[10] Kim S, Lim JD, Lockley MG, Xing L, Choi Y (2017). Korean trackway of a hopping, mammaliform trackmaker is first from the Cretaceous of Asia. Cretaceous Research.

[11] Lockley MG, Foster J (2003). Late Cretaceous Mammal tracks from North America. Ichnos.

[12] Stanford R, Lockley MG, Weems R (2007). Diverse dinosaur dominated ichnofaunas from the Potomac Group (Lower Cretaceous) Maryland. Ichnos.

[13] Contessi M (2013). First report of mammal-like tracks from the Cretaceous of NorthAfrica (Tunisia). Cret. Res..

[14] Marzola, M. *et al*. *Comparative anatomy and systematics of Cretaceous mammal tracks of Angola*. *13th Annual Meeting of the European Association of Vertebrate Palaeontologists - EAVP Opole*, *Poland: European Association of Vertebrate* (2015).

[15] Marzola, M. *et al*. Early Cretaceous tracks of a large mammaliamorph, a crocodylomorph, and dinosaurs from an Angolan diamond mine. *Journal of Vertebrate Paleontology*, *Program and Abstracts* **181** (2014).

[16] Geggel, L. Ancient animal footprints found at Diamond Mine in Angola. *Live Science* (Nov. 5, 2014).

[17] Mateus O (2017). Angolan ichnosite in a diamond mine shows the presence of a large terrestrial mammaliamorph, a crocodylomorph, and sauropod dinosaurs in the Early Cretaceous ofAfrica. Palaeogeogr, Palaeoclimatol. Palaeoecol..

[18] Lockley MG, Matthews NA, Breithaupt B (2017). A new mammal track from the Laramie Formation (Maastrichtian) at the Fossil Trace locality, Golden Colorado. Cret. Res..

[19] Sarjeant WAS, Thulborn RA (1986). Probable marsupial footprints from the Cretaceous sediments of British Columbia. Canadian J. Earth Sci..

[20] McCrea, R. T. *et al*. A review of vertebrate track-bearing formations from the Mesozoic and earliest Cenozoic of western Canada with a description of a new theropod ichnospecies and reassignment of an avian ichnogenus, In *Fossil Footprints of Western North America* (eds Lockley, M. G. and Lucas, S. G.) N*ew Mexico Mus*. *Nat*. *Hist*. *Sci*. *Bull*. **62**, 5–93 (2014).

[21] McCrea, R. T. & Sarjeant, W. A. S. New ichnotaxa of bird and mammal footprints from the Lower Cretaceous, (Albian) Gates Formation of Alberta, in *Mesozoic Vertebrate Life* 453–478 (eds Tanke, D & Carpenter, K) (2001).

[22] Stanford R, Weems RE, Lockley M (2004). A new dinosaur ichnotaxon from the Lower Cretaceous Patuxent Formation of Maryland and Virginia. Ichnos.

[23] Stanford R, Weishampel DB, Deloeon VB (2011). The first hatchling dinosaur reported from the Eastern United States: *Propanplosaurus marylandicus* (Dinosauria: Ankylosauria) from the early Cretaceous of Maryland. USA J. Paleo..

[24] Glaser JD (1969). Petrology and origin of Potomac and Magothy (Cretaceous) sediments, middle Atlantic Coastal Plain. Maryland Geological Survey Report of Investigations.

[25] Grybos M, Davranche M, Gruau G, Petitjean P, Pédrot M (2009). Increasing pH drives organic matter solubilization from wetland soils underreducing conditions. Geoderma.

[26] Gill-Frerking H, Healey C (2011). Experimental Archaeology for the interpretation of taphonomy related to Bog Bodies: lessons learned from two projects undertaken a decade apart. Yearbook of Mummy Studies.

[27] Kim J-Y, Lockley MG (2012). New sauropod tracks (*Brontopodus pentadactylus* ichnosp. nov.) and from the Early Cretaceous Haman Formation of Jinju area, Korea: implications for sauropods manus morphology. Ichnos.

[28] Kümmell SB, Frey E (2014). Range of Movement in Ray I of Manus and Pes and the Prehensility of the Autopodia in the Early Permian to Late Cretaceous Non-Anomodont Synapsida. Farke AA, ed. PLoS ONE..

[29] Peabody FE (1955). Taxonomy and the footprints of tetrapods. J. Paleo..

[30] Hunt & Lucas SG (2007). Tetrapod Ichnofacies: a new paradigm. Ichnos.

[31] Lockley MG (2007). A Tales of two ichnologies: the different goals and potentials of invertebrate and vertebrate (tetrapod ichnotaxonomy and how they relate to Ichnfacies analysis. Ichnos.

[32] Leonardi G (1981). Ichnological rarity of young in North East Brazil Dinosaur Populations. Annals Acad Brasil Ciencias.

[33] Lockley, M. G. *Tracking Dinosaurs* [238] Cambridge University Press. (1991).

[34] Hu YM, Wang YQ, Luo Z (1997). & Li. A new Symmetrodont mammal from China and its implications for mammalian evolution. Nature.

[35] Ji Q, Luo ZX, Ji SA (1999). A Chinese triconodont mammal and mosaic evolution of the mammalian skeleton. Nature.

[36] Ji Q (2002). The earliest known eutherian mammal. Nature.

[37] Kielan-Jaworowska. Z. Evolution of the therian mammals in the late Cretaceous of Asia. Part II. Postcranial skeleton in *Kennalestes* and *Asioryctes*. In Z. Kielan-Jaworowska (ed.) Results of the polish Mongolian Paleontological Expeditions. Part VII. *Paleontol*. *Pol*. **37**, 65–83 (1977).

[38] Kielan-Jaworowska. Z. Evolution of the therian mammals in the late Cretaceous of Asia. Part III. Postcranial skeleton in Zalambdalestidae. In Z. Kielan-Jaworowska (ed.) Results of the polish Mongolian Paleontological Expeditions. Part VII. *Paleontol*. *Pol*. **38**, 3–41 (1978).

[39] Carroll, R. L. *Vertebrate Paleontology and Evolution*. W. H. Freeman Co. 698 (1998).

[40] Alexander, R. Mc. N. Estimates of speeds of dinosaurs. *Nature* **261**, 129–130 (1976).

[41] Thulborn, R. A. *Dinosaur Tracks*. Chapman Hall, London 210 (1990).

[42] Lockley MG, Milner ARC (2014). The ichnotaxonomy of hopping vertebrate trackways from the Cenozoic of the western USA. New Mexico Mus. Nat. Hist. Sci. Bull..

[43] Lockley MG, Matsukawa M, Li J (2003). Crouching theropods in taxonomic jungles: ichnological and ichnotaxonomic investigations of footprints with metatarsal and ischial impressions. Ichnos.

[44] Gierliński GD, Lockley MG, Niedźwiedzki G (2009). A distinctive crouching theropod trace from the Lower Jurassic of Poland. Geol. Quart..

[45] Milner ARC, Harris JD, Lockley MG, Kirkland JL, Matthews NA (2009). Bird-like Anatomy, Posture, and Behavior Revealed By an Early Jurassic Theropod Dinosaur Resting Trace. PLoS One, PLoS ONE.

[46] Murie, O. J. *A Field Guide to Animal Tracks*. Houghton Miflin Co. Boston. 2^nd^ Ed 375 (1974).

[47] Halfpenny, J. *A field Guide to Mammal Tracking in Western America*. Johnson Books, Boulder, Colorado 161 (1986).

[48] Lockley, M. G., Hunt, A. P., & Meyer, C. Vertebrate Tracks and the Ichnofacies Concept: Implications for Paleoecology and Palichnostratigraphy, Pp. 241–268, In *The Paleobiology of Trace Fossils*, 241–268 (ed. Donovan, S.)Wiley & Sons Inc. (1994).

[49] Krapovickas V, Mángano G, Buatois L, Marsicano CA (2016). Integrated Ichnofacies models for deserts: Recurrent patterns and megatrends. Earth Sci. Reviews.

[50] Lockley MG, Fanelli D, Honda K, Houck K, Matthews NA (2010). Crocodile waterways and dinosaur freeways: implications of multiple swim track assemblages from the Cretaceous Dakota Group, Golden area, Colorado. New Mexico Mus. Nat. Hist. Sci. Bull..

