# Supplementary material for: A diverse mammal-dominated, footprint assemblage from wetland deposits in the Lower Cretaceous of Maryland
Source: Sci Rep. 2018 Jan 31;8:741. doi: 10.1038/s41598-017-18619-w (PMC5792599; doi:10.1038/s41598-017-18619-w)
Supplement: Supplementary file 1 — Supplementary Information [file 41598_2017_18619_MOESM1_ESM.pdf]

A diverse mammal-dominated, footprint assemblage from wetland deposits in the Lower Cretaceous of Maryland

Ray Stanford[1], Martin G. Lockley[2]*, Compton Tucker[3], Stephen Godfrey[4], Sheila M. Stanford[1]

[1]*NASA/Goddard Space Flight Center, Greenbelt, Maryland 20771 USA* raystanford38@gmail.com, [2]*Dinosaur Trackers Research Group, Campus Box 172, University of Colorado Denver, PO Box 173364, Denver Colorado 80217-3364, USA* Martin.Lockley@UCDenver.edu., * communicating author [3]*Earth Science Division Code 610.9, NASA/Goddard Space Flight Center, Greenbelt, Maryland 20771 USA,* [4]*Calvert Marine Museum, 14200 Solomons Island Road, Solomons, Maryland 20688 USA*, and *National Museum of Natural History, Smithsonian Institution, Washington, DC, 20560, USA* [1]*NASA/Goddard Space Flight Center, Greenbelt, Maryland 20771 USA sheilastanford@outlook.com*

Supplementary Information

Respective supplementary Information figures and tables are labelled as Figs. SI 1-SI 3 and Tables SI 1-SI 3. References cited here are included in main text.

Excavation methods

The senior author discovered the specimen at the National Aeronautics and Space Administration (NASA) Goddard Space Flight center (GSFC) facility in Greenbelt, Maryland in 2012. A small portion of the *in situ* track-bearing slab was exposed near the surface (Fig. 2A) and recognized by the senior author because a large track, (the "discovery track") was visible. The slab, in an area soon to be disturbed by construction, was uncovered to reveal a red, iron-stained surface about 2.40 m x 1.00 m in maximum length and width, and an estimated total area of ~1.90 m. In order to extract the track-bearing block with minimum damage, the sandstone around it was cut away to a depth of at least 50 cm (Fig. 2B-C), and the surface was molded in polyurethane rubber (Fig. 2D). After removal the surface was then covered with protective material before being encased in a Plaster of Paris jacket. This was the only hitherto-described *in situ* exposure of track-bearing outcrop available to help put the track-bearing slab in its sedimentary geology context.

Following the jacketing and excavation of the 50 cm thick block, it was removed to the GSFC Earth Science Division for safekeeping. Hand samples of the slab, including a piece from the actual surface and another from the sandstone about 40 cm below, were taken for petrographic analysis. A fiberglass replica of the slab surface (Fig 3A) was made from the rubber mold, and used for multiple purposes, including making of a master map (Fig. 3B). The replica was inspected closely with low angle light in order to identify small tracks. These were highlighted by application of fine sand, held in place by a weak soluble glue, which can easily be removed. Large photographs of the slab were taken before and after the application of the fine sand and used as the basis for making maps of the surface. The simplest mapping method was tracing of track outlines on clear acetate film laid over the large photographs Fig. 3B. The accuracy of the tracings was checked by constant reference to the replica. Multiple overlapping photographs were also taken for the purposes of future photogrammetric analysis.

Morphometrics of theropod, pterosaur and mammal tracks on the GSFC-VP1 specimen.

The map shown in main text Figure 3B is used here (Fig. SI 1) with labels, as a key to identifying individual tracks of in theropod trackways (prefix T), pterosaurs (prefix p) and mammals (prefix m). Measurements for tracks in both these categories are given in Tables SI 1 and SI 2 respectively. Note that most theropod tracks occur in trackway sequences (T1 – T4). Mammal tracks do not occur in continuous trackways but in some cases occur in left and right pairs indicative of sitting behavior. Likewise pterosaur tracks do not occur in trackways but in some cases occur with inferred feeding traces.

Supplementary Fig. S1.

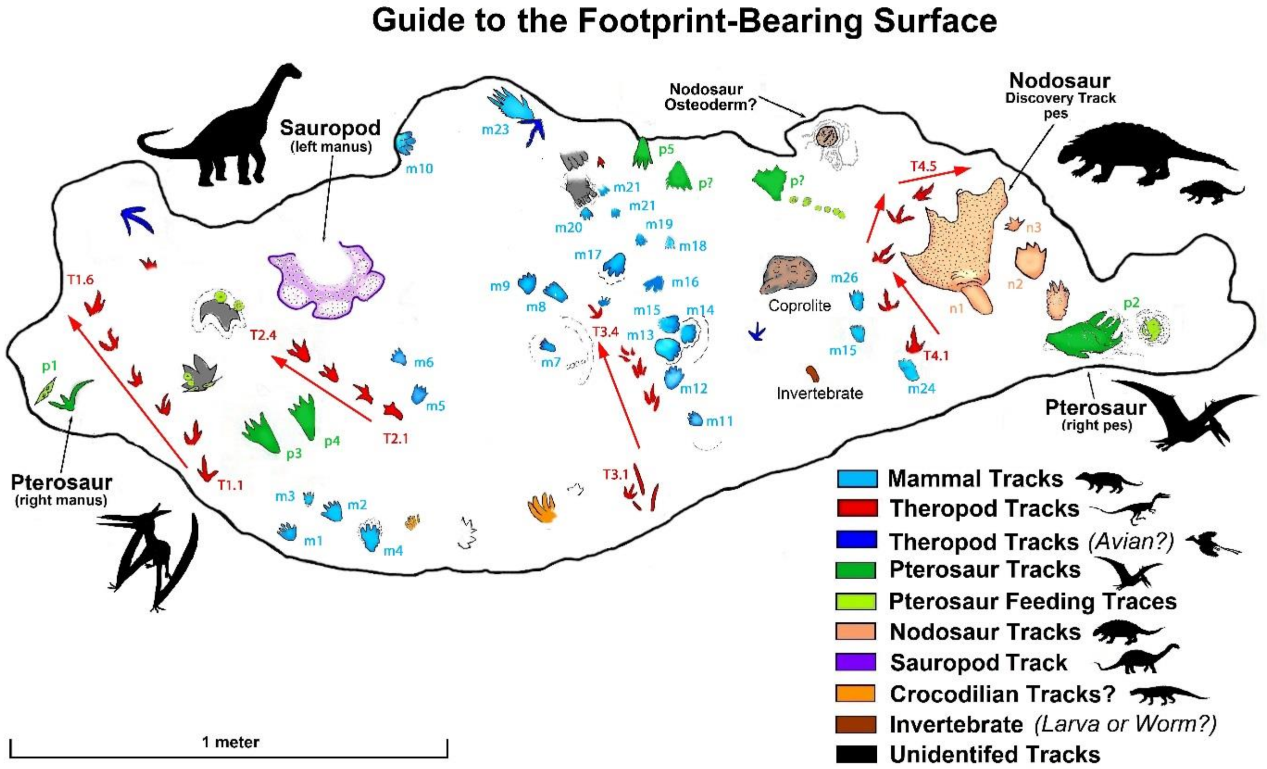

Supplementary Fig. SI 1. Key to tracks on GSFC-VP1 specimen shown in Fig 3. Theropod trackways (T), pterosaur (p) and mammal (m) track designations correspond to measurements in Tables SI- 2. Map made by the authors from original tracings and compiled in Adobe Photoshop SC6 .

Supplementary Table SI 1.

	Length L	Width W	L/W	Step	Stride	comments	
T1.1	5.9	4.9	1.20	-	-	Trackway curves to right	
T1.2	6.2	3.9	1.59	8.8	16.9		
T1.3	5.2	3.3	1.58	8.8	18.9		
T1.4	5.5	4.2	1.31	10.4	19.8		
T1.5	6.2	5.2	1.19	10.4	18.9		
T1.6	6.2	4.9	1.27	9.1	-		
**Means for T1**	**5.87**	**4.40**	**1.33**	**9.5**	**18.6**	Speed 0.22 m/s =0.80 km/hr	
T2.1	5.2	3.6	1.44	-	-	Trackway straight	
T2.2	5.2	5.2	1.00	8.1	16.6		
T2.3	4.9	3.6	1.36	8.5	18.5		
T2.4	6.8	4.9	1.38	10.1	-		
**Means for T2**	**5.60**	**4.32**	**1.30**	**8.9**	**17.6**	**Speed 0.21 m/s = 0.75 km/hr**	
T3.1	3.9	3.6	1.08	-	-	Track sequence not clear	
T3.2	6.2	3.9	1.59	-	-		
T3.3	4.6	3.9	1.18	-	-		
T3.4	3.9	2.9	1.34	-	-		
T3.5	4.6	3.6	1.28	-	-		
**Means for T3**	**4.64**	**3.58**	**1.30**	**-**	**-**		
T4.1	6.2	3.9	1.59	-	-	Trackway curves to right	
T4.2	6.2	4.6	1.35	10.4	-		
T4.3	5.9	3.9	1.51	10.4	18.9		
T4.4	4.6	4.6	0.78	10.4	19.2		
T4.5	5.9	5.9	1.51	8.1	16.9		
**Means for T4**	**5.76**	**4.44**	**1.30**	**9.8**	**18.3**	**Speed 0.22m/s =0.80 km/hr**	
**T5.1**	**2.9**	**2.6**				**only one small track**	

Supplementary Table SI 1. Morphometric parameters for small theropod trackways T1-T4 and isolated track T5 from specimen GSFC-VP1. See text for details. Step and stride data from trackways T1, T2 and T4 allow speed estimates using the formula: $v = 0.25g^{0.5} \times SL^{1.67} \times h^{-1.17}$ [40] where v = velocity, g = acceleration due to gravity, SL = stride length and h = hip height, estimated as 4.5 x footprint length[41]**.**

Supplementary Table SI 2.

Track number	Length (L)	Width (W)	L/W	Comments
m1	4.9	4.9	1.00	Morph A: *Sederipes goddardensis* holotype paired with m2
m2	4.9	4.9	1.00	Morph A: *Sederipes goddardensis* holotype paired with m2
m3	3.2	2.3	1.39	
m4	6.8	4.4	1.54	Compare with m8
m5	5.9	4.6	1.28	
m6	3.9	3.6	1.08	
m7	3.6	2.6	1.38	Morphotype: B
m8	5.5	3.9	1.41	Compare with m4
m9	4.6	4.6	1.00	Morphoptype A: 5-toed
m10	(5.5)	4.6	(1.20)	Morphotype A?
m11	(3.9)	3.6	(1.08)	
m12	4.9	4.9	1.00	Morphoptype A
m13	6.2	4.6	1.35	Morphoptype A: right footprint paired with m14
m14	(4.2)	4.6	(0.91)	Morphoptype A: 5-toed left footprint paired with m15
m15	-	-	-	Morphoptype A
m16	5.5	5.5	1.00	?Morphotype A: left footprint paired with m17
m17	5.2	5.5	0.95	?Morphotype A: right footprint paired with m16
m18	3.9	3.5	1.11	
m19	3.3	2.9	1.14	
m20	3.3	2.9	1.14	
m21	-	-	-	
m22	2.3	2.6	0.88	
m23	11.4	5.9	1.93	Morphotype C
m24	5.5	4.2	1.31	
m25	-	-	-	
m26	5.5	3.6	1.53	5-toed

Supplementary Table SI 2. Morphometric measurements for mammal footprints from the GSFC-VP1. See text for details.

Tetrapod trackway evidence for walking, running, hopping and sitting.

It is well known that trackways of walking or running quadrupeds and bipeds are represented by sequential, alternating left and right footprints (manus and pes), which may overlap in the case of quadrupeds. The main difference between trackways indicative of walking and running, which make up the vast majority of all tetrapod trackways in the track record, is simply the length of step (pace) and stride (two steps), which in turn allow speed estimates (Table SI-1). A much rarer form of locomotion is the hopping gait, recognized for only a few Mesozoic mammals[1,4,10], Cenozoic mammals and frogs[42].

There are a few examples of trackways which indicate that a bipedal animal switched from a walking to a standing, sitting or crouching posture. The best examples of such sitting or crouching traces, have, until now, come from the study of the trackways of bipedal theropod and ornithopod dinosaurs[43, 44, 45]. In these cases the alternating pattern of the walking trackway changes to one in which the left and right feet are placed side by side in symmetrical arrangement, about the trackmaker's parasagittal midline, often with the metatarsal traces impressed behind the footprint as the animal crouches. In some cases the front (manus) footprints are registered as the animal puts its front feet on the ground. No such sitting or crouching traces are known for habitually quadrupedal dinosaurs.

Until now we are unware of any reports of paired tracks which represent a mammal in a sitting posture: i.e., sitting on its haunches, even though such postures are commonly seen among extant rodents and small carnivores. Studies of hopping Cenozoic mammals[42] reveal mostly quadrupedal progression, albeit with larger hind feet registering larger footprints than the front feet. One possible sitting trace was reported in the case of the type of *Musalitpes longidigitus* where a pair of side-by site-pes tracks occur without the associated manus traces typical of the much more common *Musaltipes occidens*. It is likely that the paired configuration of the former track type (*M. longidigitus*) represents part of the trackway of a hopping individual. However, it is obvious that hopping animals would often come to rest in a sitting configuration with pes tracks side by side in symmetrical arrangement. We therefore argue that the previously illustrated pair of symmetrical pentadactyl mammal tracks[12] together with other pairs herein assigned to the new ichnospecies *Sederipes goddardensis*, represent the first unequivocal example of the tracks of a Mesozoic mammal in a typical sitting posture.

Surprisingly, despite the many field guides[46,47] in which the trackways of extant mammal species are illustrated, there are virtually no illustrations of simple sitting traces, even within trackways that otherwise show walking, running or hopping progression. This may be selective bias on the part of the authors of these guides who wish to show the traces of continuous locomotion, rather than "sedentary" sitting postures. However, from an ichnological viewpoint, sitting is a behavior which results in a distinctive trace[43, 44, 45] that is just as significant as walking, running or hopping. It may be inferred that prolonged sitting could result in subtle sinking of the feet into the substrate, at least under certain substrate conditions, so as to make a trace that is deeper, and perhaps better defined and better preserved.

Comparison between Cretaceous and extant mammal track morphotypes.

In previous studies of the Patuxent ichnofauna, mammal tracks were tracks mentioned and illustrated only briefly[12]. As explained in the main text, the pairs of tracks illustrated here (Fig. 6A, 7A) appear to be a left and right pairs which we designated as morphotype A: the holotype and a paratypes of *S. goddarensis* respectively. Also note the comparison between track m23 representing morphotype C (Fig. 6I, 7C) and the track of extant musk rat (Fig. SI 2A). Another striking comparison is between this track (M23) and the track of the mountain beaver (*Aplodontia rufa*) which is not a true beaver but a squirrel relative, sometimes considered a living fossil due to primitive morphological characteristics (Fig. SI 3B)

Due to the great abundance of tracks of small, extant mammals, in comparison with Cretaceous tracks with similar morphologies, comparisons are possible. However, it must be noted that there are manyconvergences and similarities between tracks made by different groups. Moreover, in the case of isolated tracks it may be difficult to distinguish hind and front footprints.

Supplementary Figure SI 2.

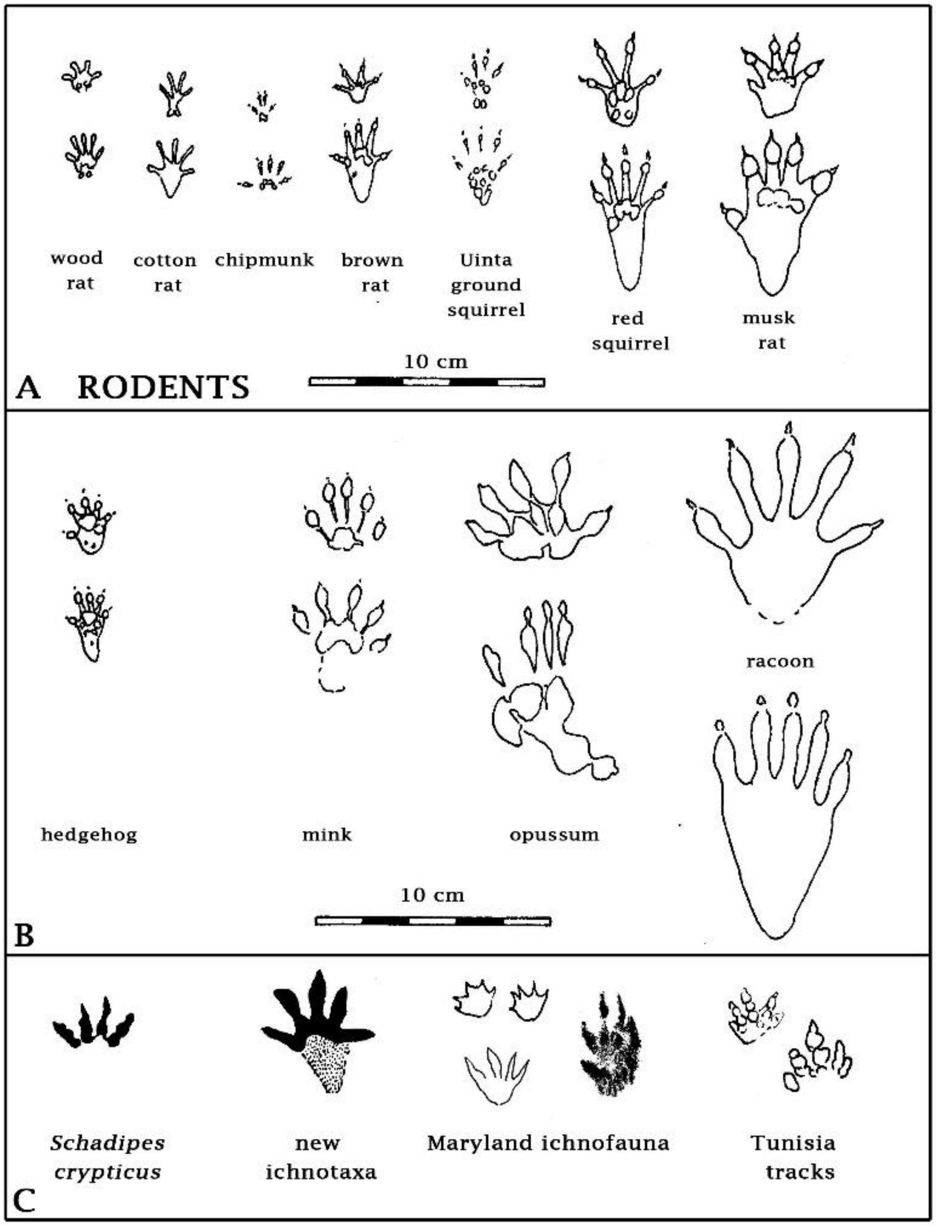

Supplementary Figure SI 2. A: tracks of extant rodents. Note four toed manus and five toed pes, B: tracks of miscellaneous extant placental and marsupial mammals, including small insectivores and carnivores. Note both manus and pes are five-toed. C: *Schadipes* from Colorado[11] (left), beside new unnamed morphotype from the same locality[18], Maryland[12] (compare with SI-Fig 3-1) and Tunisia[13]. Compare with Fig. 7. Outlines made by the authors from original tracings and compiled in Photoshop SC6 .

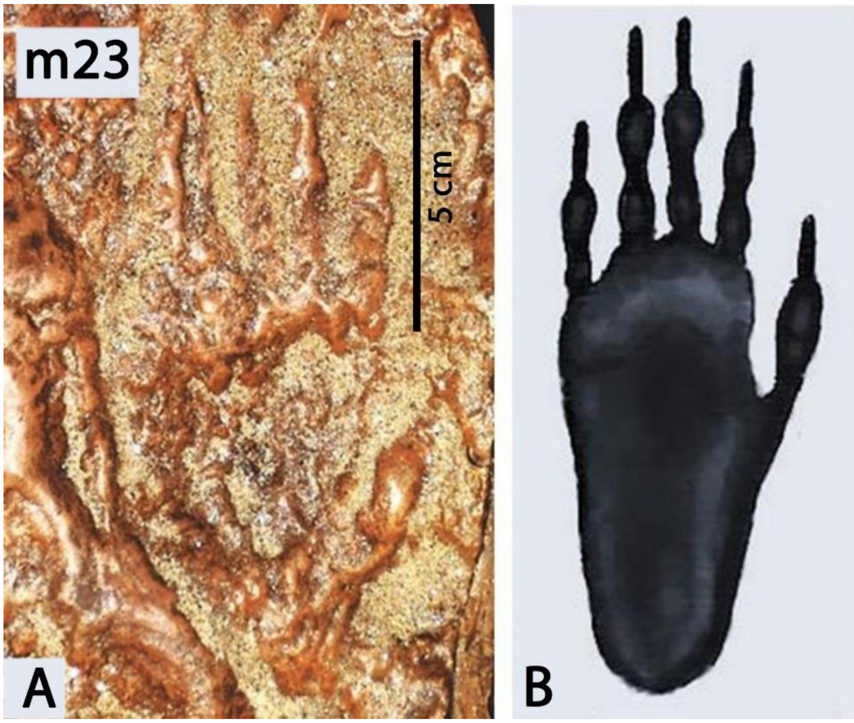

Supplementary Figure SI 3 A: Patuxent large mammalimorph track m23, compared with track of the "mountain beaver" (*Aplodontia rufa*)[47]. Photo and outline track from tracings made by the authors and compiled in Photoshop SC6 .

Ichnofacies context of Patuxent ichnofauna

Vertebrate ichnofacies were first defined in 1994[48] with limited reference to analogies with invertebrate ichnofacies, or archetypal invertebrate ichnofacies. Since then there have been attempts to distinguish consistently between local or regional ichnocoenoses and larger global or Archetypal "tetrapod" ichnofacies[30], which proponents argue should be limited to a small number, analogous to archetypal invertebrate ichnofacies. Others[31] have argued that vertebrate ichnofacies, at least most ichnofacies, are not obviously or necessarily analogous to invertebrate archetypal ichnofacies. In short, a big part of the debate revolves around whether, tetrapod ichnofacies can be subsumed into a small number (5) of categories (Table SI 3) on the basis of partly theoretical considerations about analogies with archetypal invertebrate ichnofacies, or whether the global distribution of vertebrate or tetrapod ichnofacies falls naturally into a greater number of categories. The debate has something of a "lumper-splitter" flavor with the lumpers arguing that many previously named ichnofacies, created by splitters, are in fact more localized "ichnocoenoses." The existence of labels such as sub-ichnofacies blurs the ichnofacies-ichnocoenosis distinction making the arguments semantic, conceptual and, as yet, not firmly resolved.

Supplementary Table SI 3

Archetypal ichnofacies	Predominant trace fossil types	Constituent ichnocoenoses (and age)	Inferred environment
Chelichnus	low diversity (4 types)<br>Synapsid tracks	*Chelichus* (Perm)<br>*Laopurus* (Perm)<br>*Brasilichnium* (Tr-Jur)	Eolian dune facies
Batrachichnus	Medium-high diversity,<br>4-8 types, mostly quadrupedal carnivores	*Batrachichnus* (Carb-Perm) with 3 sub ichnocoenosis	Tidal Flat-fluvial plain
Brontopodus	Medium high diversity 4-8 types mostly quadrupedal herbivores with >10% carnivores	ornithischian (*Ceratopsipes* & *Caririchnium*) and saurischian (*Brontopodus*) Coastal plain, clastic and carbonate (Jur-Cret).<br>Ironstone ichnocoenosis (Cret)	Coastal plain, clastic and carbonate shoreline
Grallator	Medium-high diversity (5-8 types) mostly bipedal avian and non-avian theropods	*Avipeda* (Cenozoic)<br>*Eubrontes* (Jurassic)<br>*Jindongornipes- Koreanaornis* (Cret)	Lacustrine margin
Charachicnos	Scratch marks and fish swim traces	*Characichnos* and<br>*Hatcherichus* (Jurassic)	Shallow lacustrine

Supplementary Table SI 3. Summary of proposed 5-fold classification of tetrapod ichnofacies[30] and constituent ichnocoenoses named since 1994, with Patuxent ironstone ichnocoenosis (in red) tentatively placed in the context of the scheme.

The debate continues, with some common agreement that the vertebrate and invertebrate ichnofacies of eolian or dune facies (Table SI 3), the first of five proposed archetypal categories[30], are more or less co-extensive: i.e., the ichnofacies models for deserts[49] regard the tetrapod ichnofacies label *(Chelichnus* ichnofacies) as similar and coextensive with the invertebrate ichnofacies label (*Entradichus-Octopodichnus* ichnofacies), as already agreed by various workers [30,,31]. For the purposes of clarification and comparison the archetypal tetrapod ichnofacies of Hunt and Lucas[30] are tabulated below (Table SI 3) in simplified form as a point of comparison for placing the Patuxent ichnocoenosis in its ichnofacies context.

The archetypal tetrapod ichnofacies scheme summarized in Table SI 3 proposes that all non-eolian and non-subaqeous lacustrine ichnofacies can be subsumed in three archetypal ichnofacies: *Batrachichus* (Late Paleozoic), *Brontopodus* (Late Mesozoic) and *Grallator* (Jurassic-Cenozoic) each with about three constituent ichnoconoses, some of which were previously labelled as ichnofacies[30,31,48]. We do not here evaluate the merits of this scheme except to note that it is very broadly conceived, and that in some places traces of more than one ichnofacies interpenetrate[50]. We present this scheme, not to endorse or refute it, but only to place the Patuxent ironstone ichnocoenosis in context. We infer that the authors of this scheme[30] would likely agree that the ironstone ichnocoenosis is an ichnocoenosis, not an ichnofacies, and that it would best be subsumed within the so called *Brontopodus* ichnofacies associated with coastal plain settings. However as noted in the main text the Patuxent ichnocoenosis is significantly different in composition (diversity) and facies association from the characteristics of the *Brontopodus* ichnofacies as defined, as well as from its other constituent ichnocoenoses. This is evidently due to the near optimal preservation of small tracks, rarely or never found in the other ichnocoenoses so far identified. Thus, we argue that the Patuxent ichnofauna can be tentatively placed as shown in table SI 3, in its broad paleoenvirnomental context as a newly recognized "ironstone ichnocoenosis." As noted in the main text this ichnofauna (ichnocoenosis) has implications for more general questions of incomplete preservation in other ichnocoenoses, and how they are defined.
